# Domestic Summary

**Published:** 1839-01-26

**Authors:** 


### DOMESTIC SUMMARY.

*Case of Poisoning from Pork.*—Dr. Waller R. Dupuy, of Carrollton, Illinois, has communicated to us an interesting case of fatal poisoning from the use of fresh pork. The diagnosis Dr. D. was unfortunately not able to verify by an autopsic examination; but from the symptoms reported, we are led to the conclusion that the cause of death was the poisonous action of pork upon the stomach and bowels. Several cases of similar effects of this article are on record in British Journals, and a detailed account of such a case is contained in the first number of the first volume of this Journal, by Dr. Pepper, of Philadelphia.

"On the 7th of December last, I was called, at 5 o'clock, P. M., to see a patient, who had been vomiting an hour, perhaps, when I saw him; he threw nothing off his stomach but a glairy fluid. He had an obtuse diffused pain throughout the abdomen, which appeared to be seated rather in the periphery, than centre of the cavity of the abdomen.

"For many months previous to the above date he had been labouring under chronic gastro-enteritis, frequently liable to colic, from one attack of which, some four or six weeks previously, I had relieved him; and he was often thus disturbed by any incidental irritating ingesta, though he still pursued his occupation of shoemaking. The day previous to that on which he was taken, he had eaten freely of *fresh pork;* and at noon of the day of his attack, he partook bountifully of the same unwholesome diet. He then immediately exposed himself, for two or three hours, to a very cold and humid atmosphere, in the open forest,—and while there, I am informed, he ate some grapes, which were astringent in character. All the above causes simultaneously in operation, produced what we might suppose, in one whose gastric surface had long been in a state of irritation. When I first saw the patient, three hours after he was attacked, already he was so distended as to forbid full emesis, which I at first endeavoured to effect; but finding it impracticable, I then resorted to enemata, various and repeated, but all to no purpose. Finding I could not evacuate the alimentary canal, either per orem or per anum, (although I introduced a tube into his rectum, some way up into the sigmoid flexure of the colon,) I gave him opiates and antispasmodics, but without benefit. The organic lesion went on; distension increased rapidly; great tympanitis was present; and, in less than four hours from my first attention to the case, and seven hours from the first of the attack, so great was the distension, that the abdomen was almost as incompressible as a deal board. From the epigastrium, down, there was a total paralysis of sensation and motion. Three hours before he died, a piece of ice was not felt when placed on the surface of his body below the gastric region—nor was muscular motion possible; yet, until about an hour before dissolution, there was no disturbance of the intellect, and, throughout, there was but little morbid animal sensibility; no convulsion, but a complete tetanic rigidity of the muscles of the abdomen and lower limbs. His rectum finally protruded several inches, so as to extrude the glyster pipe completely. We lastly gave him a strong decoction of tobacco; but it was impossible for him to vomit, and he could barely spit it up,—so entirely had all muscular phenomena disappeared an hour before he died, which occurred in nine hours from the attack. I regret that no autopsy was permitted.

"From the rapid termination of this case, (nine hours after the attack,) and the previous good health of the patient, I am induced to reject the idea that this case could have been one of perforation of the intestines. A case of perforated bowel usually lingers until death takes place from peritoneal inflammation, which rarely happens in less than from one to three days. There is, besides, usually much more pain than was evinced in this case. I can scarcely suppose that the grapes, which the patient partook of, could have been poisonous berries; the taste is quite dissimilar, and could easily have been detected. I am inclined to adopt the opinion, that the principal cause of death was the deleterious action of the pork upon a stomach and bowels in a condition of chronic irritability. The patient was, moreover, hereditarily liable to severe attacks of colic, and a tolerably free partaker of ardent spirits, though not a drunkard. A combination of causes probably contributed to death."

